# Progress on Nutrient Composition, Meat Standardization, Grading, Processing, and Safety for Different Types of Meat Sources

**DOI:** 10.3390/foods10092128

**Published:** 2021-09-09

**Authors:** Nelson Huerta-Leidenz

**Affiliations:** Department of Animal and Food Sciences, Texas Tech University, P.O. Box 42141, Lubbock, TX 79409-2141, USA; nelson.huerta@ttu.edu; Tel.: +1-956-250-4337

## 1. Introduction

Beef contains a plethora of healthy nutrients and it is the highest valued livestock-based food product. However, other meats (such as pork and poultry) and co-products of the meat industry can also be nutrient-dense with advantageous sensorial and technological qualities. The current standardization and grading schemes can assist in describing meats for their eating quality and/or fabrication yield, but more innovative, objective technologies are much needed to improve the segregation of the heterogeneous supply of carcasses and cuts into more homogeneous groups as regards quality and/or yield. In terms of food safety, while there have been significant improvements in reducing foodborne illnesses in the meat industry, the morbidity and mortality attributed to *Salmonella*, *E. coli* O157: H7, *Listeria monocytogenes, Campylobacter jejuni*, and other pathogens remain an issue with serious socioeconomic impacts. There is, therefore, a need to evaluate antimicrobials and technologies to assist in mitigating these recurrent problems in public health. This Special Issue of *Foods* was designed to cover scientific and technological advances in selected topics of global importance for the progress of the livestock and meat industries. Therefore, in this Special Issue, we have included contributions that encompass key current research on nutrient composition, instrumental meat grading, and food safety. This collection of scientific articles and reviews is fundamentally a profile of a much broader perspective of animal and food sciences applied in trans-cultural settings.

## 2. A Summary of the Research in this Special Issue

The summary and comments about the papers published in this Special Issue deal mostly with findings directly related to the themes of progress on nutrient composition, meat standardization, grading, and safety for different types of meat sources. While included in some of the research, findings related to sensory quality and other aspects outside of the Special Issue themes are not addressed in this preface in order to focus specifically on the targeted topics. However, we cordially invite the readers to discover the entire body of knowledge compiled in these articles, which extends beyond the defined parameters of this Special Issue.

### 2.1. Progress on Nutrient Composition

Two state-of-the-art extensive reviews are published on this topic, one [1] informing on the genetic and nutritional strategies available to enhance the nutritional quality of red meat (168 reviewed papers), and the other [2] attempting to characterize the quality and nutrient composition of meat produced in the tropics (147 reviewed papers). The original articles that dealt with the nutrient composition of meats and co-products, separately or concurrently with aspects of eating quality, were the results of research carried out in various countries, in different species and environments, including Chile [3] (the effect of supplementing pigs with brown seaweed on quality traits and nutrient composition of pork), Canada [4] (the impact of feedlot diets containing various levels of barley on the nutritional quality of *Bos taurus* beef), Spain and USA [5] (a comparison of the veal produced by bullock calves in the Pyrenees [PGI-Certified *Ternera de Navarra* (*CTNA*)] and US Angus certified beef), Colombia [6] (characterization of the lipid profile of visceral fats by-products of chicken), and Venezuela [7] (multivariate relationships between nutrient composition and carcass characteristics of *Bos indicus*-type cattle raised on pasture in a tropical environment).

Juárez et al. [1] update us on the available genetic and nutritional strategies to enhance the nutritional quality of red meat (beef, lamb, and pork). This subject is particularly pertinent today, given the numerous studies suggesting that red meat consumption may have negative effects on human health and the environment. This review identifies information gaps on the evaluation of genetic parameters related to meat composition, as well as multiple bioethical challenges linked to new trends in genetic engineering, while also demonstrating that much progress has been achieved regarding the dietary manipulation of the nutrient content of meat. The authors [1] note that most studies used approaches that independently assess the impact of genetics or nutritional strategies, but few explored the interactions between these two factors.

The most notable findings suggesting potential benefits in genetic manipulation [1] were: (a) differences between breeds or heritability values reported for concentrations of certain vitamins (E and B) and minerals (copper, total iron (myoglobin), and selenium) in beef; there are no reports on the heritability of vitamin A content in muscle, (b) genetic manipulation of zinc content in lambs and pigs, uncertain in cattle, (c) highly variable heritability values are reported for total protein content and individual amino acids, (d) there is the potential to manipulate individual amino acid concentrations by genetic and dietary means, (e) well-known differences between and within breeds in terms of total variability of intramuscular fat (IMF), with very obvious cases of genetic groups with greater levels of marbling [1], and (f) genetics not appearing as a primary factor in the accumulation and proportions of *trans* fatty acids in beef.

Regarding the potential for dietary manipulation of micronutrients, the main findings of the review by Juárez et al. [1] were: (a) pasture feeding increases the levels of vitamins or their precursors (tocopherol, b-carotene, thiamine, riboflavin, lutein, retinol, a-tocopherol, and g-tocopherol) in beef or suckling veal under grass-feeding systems, (b) there is a favorable response to the specific supplementation of vitamins (higher concentration of vitamin E in cattle and of B9 and B12 in pigs) or minerals (selenium and iodine in cattle) but not of zinc, and it is pointed out that it is easier to reduce the iron content in beef than to increase it, (c) in pigs, feed additives derived from algae have an impact on the nutrient potential of B vitamin concentrations, (d) monogastric diets are usually complemented with B vitamins, and although additional increases have a small impact on the muscle concentration of B9 and B12 vitamins, vitamin B2 levels do not seem to be affected by higher supplementation, (e) gastrointestinal–pancreatic control of zinc absorption hinders its manipulation through the diet, and (f) most studies have reported little or no effect on zinc muscle concentrations after zinc supplementation, particularly in pigs.

Jerez et al. [3], when evaluating the addition of two amounts (2 or 4%) of brown seaweed to the regular diet of finishing pigs, found that total lipids and microminerals such as Cu, Zn, and Mn decreased in the muscle of pigs fed the higher percentage of seaweed (4%), despite a small but significant increase in total ash. However, the fatty acid composition of pork was not influenced by the inclusion of the brown seaweed additive at any level. Inexplicably, these authors [3] found a higher cholesterol content in the meat of pigs fed the diet with the higher content (4%) of brown seaweed, suggesting that there are some components of algae that impact cholesterol content.

The modification of the fatty acid profile of beef by dietary means still faces the difficulty of a buffering effect of the extensive ruminal biohydrogenation which transforms unsaturated fatty acids into saturated fatty acids (SFA) [1]. However, pasture feeding and feeding management have assisted in increasing beneficial biohydrogenation intermediates. For example, supplementing ingredients rich in polyunsaturated fatty acids (PUFA), such as flaxseed co-extruded with peas, before feeding hay, instead of feeding hay and a mixture of supplements, leads to a substantial increase in vaccenic acid and conjugated linoleic acid (CLA), and these differences are related to changes in the microbial population in the rumen. According to Juárez et al. [1], those ingredients or additives in the cattle diet that modify the rumen microbiome may have a greater impact than a direct fatty acid supplementation.

Concentrate-based diets have been associated with a decrease in PUFA/SFA ratios, but, as the finishing period progresses, there is a higher conversion of SFA to monounsaturated fatty acids (MUFA), and the relative rates are influenced by breed [1]. The study conducted by Barragan-Hernandez et al. [4] in this Issue of *Foods* examined the effect of grain type in the diet (corn vs. barley vs. a barley/corn mix) on the sensory attributes, volatile compounds, and beef flavor profile of steers. The authors also examined the normalized partial profile (% of total) of fatty acids presumably responsible for variations in flavor. They [4] reported that the corn-based diet elicited a significantly lower proportion of n-3 fatty acids, a higher proportion of stearic acid (considered to have a neutral cholesterolemic effect), and a higher value of the n-6/n-3 ratio compared to the other two treatments (respective means of 8.32, 6.22, and 7.26 for the corn-, barley-, and barley/corn mix-based diets). Although significant, differences in the n-6/n-3 ratio could be considered low in magnitude and possibly irrelevant from the point of view of human health; authors [4] did not discuss this. In fact, although some authors recommend that the average value of the n-6/n-3 ratio should not be greater than 5.1, the recommendation of the WHO is that the value of this index should not exceed 10 [3], which indicates that the n-6/n-3 values for the different grain diets tested by Barragan-Hernandez et al. [4] are within the safe range.

According to Juárez et al. [1], the dietary effects observed in IMF tend to have a greater impact on the most abundant fat deposits, which is important to try to improve the lipid profile and achieve certain marketing (particularly, health) claims. In fact, these authors [1] suggest an alternative to ground beef, based on feeding a small group of animals with diets characterized by a high concentration of certain beneficial fatty acids or selecting carcasses naturally presenting this feature and then mixing the fat from those animals or carcasses with lean meat from the regular population. For their part, Peña-Saldarriaga et al. [5] with a similar proposal, pointed out the potential of using fat that can be removed with high yields as a co-product of chicken. According to their analyses, these underutilized fats contain—without significant variation due to environmental factors—a lower concentration of myristic acid (an undesirable saturate) and a greater proportion of essential polyunsaturated fatty acids (PUFA, approximately 40% of the total UFA) when compared with pork fat or beef tallow (the latter having less than 20%). The authors [5] suggest using these fats in the formulation of meat products such as sausages, replacing other sources of animal fat (for example, bird skin) commonly used in the poultry industry.

Another review and one research article deal with the composition of nutrients in beef produced in the tropics. When trying to characterize this type of meat through an extensive review of the literature, Rubio et al. [2] pointed out that, in general, (a) there is little variation in macronutrients (almost all protein content values are in the range of 20% to 24%, with some differences due to castration, genetic influences, or to the finishing on pastures vs. grain), (b) the proximate component that showed the largest variation was the IMF content that varied from 1.0% to 8.9%, but most of the literature indicates a lean beef, with <3.6% of IMF, (c) low marbling scores (IMF), which are typically observed in cattle influenced by *B. indicus*, are attributed to the reduced volume of adipocytes, and not to the quantity of cells, (d) there are few studies on the mineral content of cattle raised in tropical environments, and of note is that the feeding system (pasture with or without supplementation) had little to do with the beef mineral content, but an age effect was observed when comparing grazing cattle at 17, 19, and 24 months of age, (e) the impact of climatic conditions on the edible tissues of tropical cattle should be considered, in particular, pastures suffer a seasonal effect on their quantities and qualities, and fluctuations in feed quality can cause mineral imbalances throughout the year, and (f) there are indications that the IMF of steers with genetic predominance of *B. indicus* contains more myristic, palmitic, linoleic, and linolenic acids, but less stearic acid than their counterparts with predominance of *B. taurus*.

According to Rubio et al. [2], the complexity of the research on the impact of genetics on the fatty acid composition of tropical cattle meat is greater than previous studies suggest. In one of the few studies reported, 14 of the 43 individual fatty acids and fatty acid indices in the IMF were affected by an interaction between the genetic pool and the cattle finishing system. Of the 29 fatty acids and fatty acid indices for which the interaction was not significant, 11 were influenced by the genetic group, and 25 by the finishing system. In general, with the exceptions of cholesterol, 18: 1 *trans*-6, -7, -8, 18: 1 *trans*-12, and the 22: 5, n-3/18: 3, n-3 ratios, all fatty acids and individual indices were affected by at least one of the factors considered or by their interactions. Differences between genetic groups were lower with pasture finishing, but under grain finishing, *B. indicus* showed higher amounts of SFA and stearic acid and lower concentrations of fatty acids synthesized from linoleic and gamma-linolenic acids. In general, animals finished on grass produced meat with lower levels of IMF, *trans* fatty acids, and SFAs and higher content of CLA and long-chain PUFA (20: 5, n-3 and 22: 5, n-3). Rich diets in forage favor the growth of the fibrolytic microorganisms responsible for CLA production, and forage-fed livestock has higher concentrations of linoleic, stearic, arachidonic acids (20: 4, n-6), eicosapentaenoic (20: 5, n-3) and docosapentaenoic (22: 5, n-3) acids in the meat than animals fed with concentrates [2]. However, IMF contents are often low in grass-fed beef (<2 g/100 g of fresh muscle), and this meat cannot therefore be considered a significant source of CLA. Again, it should be noted that climatic variations in tropical regions can greatly affect the quality of the grass and hence its nutritional contributions. A review in this Special Issue [2] cautions against genetic manipulation based on a selection of zebu cattle (Nellore) with lower body fat to a given weight, because such a genetic approach would decrease the proportion of MUFA (oleic acid) in the subcutaneous fat depot, with concomitant increases in saturated fatty acids, such as stearic and other, less healthy, saturated fatty acids such as myristic and palmitic acids.

The potential of carcass traits for assisting in the prediction of beef chemical components was evaluated by Arenas de Moreno et al. [7]. In this study, the authors performed an analysis of hierarchical conglomerates and canonical correlations to explore multivariate relationships between selected traits of the beef carcass derived from cattle fed on tropical pastures and chemical components (proximate, minerals, and lipids) in *longissimus lumborum* muscle (LL). The statistical approach is demonstrated as a powerful tool to study the relationship between a selected set of carcass traits and the proximate, lipid, and mineral components, particularly when there is a certain degree of interaction between the three groups of chemical variables. The association of carcass traits and minerals was poor. However, the analyses pointed out an important relationship of backfat thickness and marbling scores with the content of total lipids and fatty acids in the LL. In their conclusions, the authors argue in favor of backfat thickness, rather than marbling, as the most feasible potential predictor for performing future regression analyses attempting to explain the variation in lipid composition of this type of livestock.

### 2.2. Progress on Meat Standardization and Grading

Product consistency and differentiation are proved tactics for succeeding in meat marketing and trade. The certification of beef carcasses serves for a series of marketing programs. Several certification programs are based on a set of specifications (that may include a breed) to make marketing statements on certain characteristics, especially quality. In the USA, these specifications go beyond the requirements required for the grades offered by the official grading system and are the basis for the different branding programs endowed by the USDA. Certified Angus Beef (CAB) is the most recognized meat branding program in the USA. On the other hand, Protected Geographical indications (PGI) commonly used in the EU are certification programs based on original, identifiable characteristics of a product derived from a specific location in order to protect its quality and reputation. These two distinct marketing strategies have the same purpose: product differentiation. Beriain et al. [7] compared the veal produced by bullock calves in the Pyrenees [PGI-Certified *Ternera de Navarra* (*CTNA*)] to US-CAB. Physicochemical and sensory traits were assessed in Spain (Navarra) and USA. The authors found noticeable contrasts (i.e., marbling, IMF, and other proximate components) which are explained not only by the animals’ distinct genetic make-up but also by their dissimilar age, sex, and management. The authors highlighted that the taste panels in the two countries agreed that the *CAB* striploins outperformed the *CTNA* samples in juiciness, tenderness, and flavor, notwithstanding the similarities between CAB and CTNA in total and soluble collagen contents.

Segura et al. [8] determined the potential of computer vision systems (CVS), namely, the whole-side carcass camera (HCC), to the rib-eye camera (CCC), and the dual-energy X-ray absorptiometry (DXA) technology, to predict the composition of wholesale cuts and carcasses of mature cows. This comparative study [8] was carried out in Canada where the classification system segregates mature carcasses (for example, cows) as Canada D, with a series of designations (Canada D1 to D4) for the types of carcass that do not count on a method for predicting the cut-out yield before fabrication. The technologies used in the study [8] provided estimation values of the total amount of tissues and a general description of the composition of the entire carcass and its primal cuts without requiring the destructive procedure of dissection. The DXA technology could be considered the gold standard for estimating carcass composition. The primary estimates of DXA, on average, had higher R^2^ values for fat (0.95), lean (0.97) and bone (0.82) than those of CVS, and even exceeded the prediction equations using all variables retrieved by the cameras (HCC and CCC). However, to date, DXA has been limited by practical restrictions in industry implementation (horizontal table scan, room temperature operation, and scan speed in minutes instead of seconds). Instead, CVS technologies (HCC and CCC cameras) are being widely implemented in the USA and Canada. According to their findings, the authors determined the feasibility of using HCC to predict the composition of carcass and wholesale cuts, and the combination of the two CVS technologies led to significant improvements in the predictions, in particular, for the lean/fat ratios, suggesting that the dual CVS approach is an alternative for improving the accuracy of predicting the composition of carcasses and primal cuts of cull cows. The assessment of different types of instrumental grading would allow not only finding out the best technology for differentiating these products in the marketplace but also identifying new opportunities for the future development of automation in the meat industry, an emerging need in the pandemic era.

### 2.3. Progress in Food Safety for Different Types of Meat Sources

Six articles are presented on food safety. One article evaluates four antimicrobials on refrigerated pork loins [9], another assesses the antimicrobial application mode (immersion vs. spray) to reduce *Campylobacter jejuni* in chicken wings [10], four deal with physical and/or chemical interventions, such as the use of UV-C solely or in conjunction with antimicrobials [11,12], refrigeration technologies (dry chilling vs. spray chilling) combined with hot water washing by bio-mapping of indicator organisms on beef striploins during storage [13], and in-plant validation of a novel aqueous ozone generation technology compared to lactic acid solutions for suppressing the growth of natural microbiota, i.e., *E. coli* O157:H7 and *Salmonella* surrogates, on beef carcasses and trimmings [14].

Antimicrobial sprays evaluated by Vargas et al. [9] on pork loins subjected to four refrigerated storage periods (1, 14, 28, and 42 days) included: cold water (control), 1,3-dibromo-5,5-dimenthylhydantoin at 225 ppm (Bovibrom-225), the same active principle as Bovibrom-225 but at 500 ppm (Bovibrom-500), chlorine dioxide at 3 ppm (Fit Fresh), and Rhamnolipid at 750 ppm (Natural Washing Solution). Initial counts did not differ between treatments, while as for after-treatment interventions, the treatment with Natural Washing Solution did not effectively reduce the counts of APC-mesophilics, APC-psychrotrophs, and coliforms (*p* < 0.01). The antimicrobials Bovibrom-500 ppm, Fit Fresh, and Natural Washing Solution were the best in maintaining reduced microbial counts when compared to control treatments of pork loins after 14 days of storage under refrigerated conditions at 0–4 °C.

Gonzalez et al. [10] inoculated surfaces of fresh, skin-on, chicken wings with 3.9 log colony-forming units [CFU]/mL) of a mixture of six *Campylobacter jejuni* strains of poultry origin. The inoculated wings were left untreated, to serve as controls, or they were treated by immersion or spray application of water, a blend of sulfuric acid and sodium sulfate (pH 1.2; SSS), formic acid (1.5%; FA), peroxyacetic acid (550 ppm; PAA), PAA (550 ppm) acidified with SSS (pH 1.2; SSS-aPAA), or PAA (550 ppm) acidified with formic acid (1.5%; FA-aPAA). All five chemical interventions were efficacious (*p* < 0.05) in reducing *C. jejuni* populations on chicken wings, with larger immediate reductions by immersion than by spraying. Acidification of PAA (550 ppm) with SSS or FA did not enhance the immediate (0 h) bactericidal effects of non-acidified PAA. However, the combination of the acidified PAA treatments and the subsequent chilled storage conditions (4 °C, 24 h) likely prevented the recovery of sub-lethally injured bacterial cells. As a result, chicken wings treated with SSS-aPAA or FA-aPAA and stored at 4 °C for 24 h showed the lowest pathogen levels.

Calle et al. [11] evaluated the use of UV-C LED light for the destruction of *Salmonella* present on chicken breast and food contact surfaces. The antimicrobial properties of UV light have been explored elsewhere, mainly for applications in liquids, contact surfaces, and packaging materials, where its effectiveness has been demonstrated. However, the most common application involves the use of mercury lamps. The growing interest in ultraviolet (UV) light was driven by its FDA approval in 1997 for surface decontamination of foods. According to the literature [11,12], several facts of UV irradiation use in food safety are reported: (a) pathogens absorb UV light, and thymine-dimers molecular lesions in the DNA are formed via photochemical reactions, ultimately leading to cell death, (b) UV light is currently used to control pathogens in water and for the decontamination of food contact surfaces and food packaging materials, (c) UV light-emitting diodes (LED) are increasingly being used as substitutes for mercury lamps, conventional sources of UV-light, for their smaller size and lesser generation of heat, (d) the emission spectrum of UV-LED can be tuned to emit UV light of specific wavelengths between 250 and 280 nm, which are the most effective at driving the photochemical reactions leading to the formation of thymine dimers, (e) UV-LED devices are more robust, durable, and safe compared to mercury lamps because they do not contain glass tubes that may break and contaminate workstations with mercury, (f) UV-C band irradiation stands out for its low cost, with no potentially hazardous chemical residues, and low carbon footprint [12].

In the USA, Calle et al. [11] have shown that UV-LED could be used to disinfect skinless chicken breast (CB) as well as food contact surfaces such as stainless steel (SS) and high-density polyethylene (HD) inoculated with *Salmonella enterica*. The greatest reductions were obtained after 180 s of exposure on HD (5.2 Log CFU/cm^2^), followed by 60 s on SS (3.5 Log CFU/cm^2^), and 900 s on CB (3.0 Log CFU/cm^2^). The best reductions were obtained when UV-C LED was applied on SS. For example, 60 s of exposure yielded 3.48, 2.05, and 1.77 Log CFU/cm^2^ on SS, CB, and HD, respectively. The porosity of surfaces appears to play a role in the effectiveness of the UV-C LED light, since bacterial cells appear to be shielded by hollow surfaces, as observed in electron micrographs.

The most typical chemical interventions to reduce *Salmonella* and other pathogens in poultry and red meat products involve the application of treatments at different steps of processing, which include the use of organic acids, inorganic compounds, chlorine-based treatments, and phosphate-based products, among other compounds [11]. However, consumers seem to have adverse opinions about the use of such chemicals in foods [11], whereas lactic acid (LA) application at a maximum concentration of 5% (*m*/*v*) is generally accepted because it does not present risks to consumer health [12]. It is known that *Listeria monocytogenes* can survive and grow in vacuum-packaged meat cuts stored at temperatures between 0 and 4 °C. In Uruguay, Brugnini et al. [12] studied the combined effect of UV-C and the application of lactic acid on the inactivation of *Listeria monocytogenes* and lactic acid bacteria (LAB) in vacuum-packaged beef. Surface response analysis indicated that a maximum log reduction for *L. monocytogenes* (1.55 ± 0.41 log CFU/g) and LAB (1.55 ± 1.15 log CFU/g) with minimal impact on meat color was achieved with 2.6% LA and 330 mJ/cm2 UV-C. This strategy could be useful to ensure beef safety and to help extend the shelf life of vacuum-packaged beef to safely reach distant markets. These two studies [11,12] further support the use of UV as a “no-touch” technology in the food industry to effectively sanitize high-touch surfaces where there may be a higher risk of meat contamination from pathogens. UV disinfecting technologies have been used for a number of years and they could be more effective with improved features in the future, given their constant innovation.

Casas et al. [13] evaluated the impact of spray and dry chilling combined with hot water treatments on the levels of microbial indicators during refrigerated storage of beef striploins at an Australian beef processing plant. A total of 200 carcasses were evaluated. Samples were taken before and after (washed samples) carcass hot wash and 24 h after subjecting the carcasses to spray vs. dry chilling. The hot water carcass wash consisted of spraying water at 85 ± 2 °C onto the surface of the carcasses. The spray chilling method consisted of continuously spraying water at 0–2 °C at 15 min intervals, during 18–24 h storage. Dry chilling consisted of 18–24 h storage in a refrigerated room at 0 °C with constant airflow, while the sprayers were turned off. The excised striploins were cut into four sections that were individually vacuum-packaged to be sampled after 0, 45, 70, and 135 days of storage and distribution under refrigeration. Aerobic plate counts (APC), enterobacteria, *Escherichia coli*, coliforms, and psychrotroph (PSY) counts were evaluated for each sample. Under the conditions evaluated in this study, the hot water carcass intervention was not found to significantly reduce APC and PSY counts compared to the no-wash treatments. Despite significantly reducing a small number of bacterial species on the surface of the carcass, washing may also redistribute the bacteria throughout the whole carcass surface and can contribute to further microbial attachment, growth, and development during prolonged storage. The authors [13] concluded that the optimal shelf life of striploins can be achieved using dry chilling air systems, which will guarantee the required 130 days of shelf life for the export of fresh, never frozen beef from Australia to the EU. The use of spray chilling schemes increases the available water for the growth of bacteria, resulting in higher growth rates of bacteria during long-term refrigerated storage and, therefore, in a reduced shelf life.

Casas et al. [14] assessed the antimicrobial efficacy of an aqueous ozone solution (Bio-Safe) and lactic acid solutions on the natural microbiota and *E. coli* O157: H7 and *Salmonella* surrogates in beef carcasses and trimmings at a commercial meat processing plant. The lactic acid operating parameters applied in the plant for this study included treatment with a 2–5% lactic acid solution sprayed at a temperature of 43–55 °C, with a spray pressure of 15 psi. The operational parameters of the ozone intervention included generators that use air oxygen molecules (O_2_) passed through a crown field, which divides them into individual oxygen atoms (O). These individual O atoms combine with an O_2_ molecule to form an ozone molecule (O_3_). After the intervention and immediate reaction with organic matter, O_3_ becomes oxygen again, without leaving byproducts or harmful waste, according to the description of the manufacturer and the patented technology developed. Ozone and lactic acid interventions significantly reduced (*p* < 0.003) bacterial counts in carcasses and trimmings. Furthermore, lactic acid further reduced APC and coliforms in trim samples as compared to the ozone intervention (*p* < 0.009). Ozone significantly reduced (*p* < 0.001) the concentration of *Salmonella* surrogates. According to the plant’s historical data, a reduction (*p* < 0.001) of presumptive *E. coli* O157: H7 in trimmings was recorded after a full year of implementing the ozone intervention. These results are very promising, since the use of ozone in combination with organic acids would allow a more efficacious, safe approach for the decontamination of beef carcasses and products. According to the authors [14], this new technology for ozone generation and its application as an antimicrobial can become an alternative that may also act synergistically with existing interventions, minimizing the risk of *Salmonella* and *E. coli* O157: H7.
